# Expression of Concern: Targeting tumor-associated genes, immune response, and circulating tumor cells in intrahepatic cholangiocarcinoma: Therapeutic potential of *Atractylodes lancea* (Thunb:) DC

**DOI:** 10.1371/journal.pone.0352929

**Published:** 2026-07-02

**Authors:** The PLOS One Editors

After this article [1] was published, the following concerns were noted:

- The article cited as Reference 53 was retracted before [1] was published.
- The article cited as Reference 17 was retracted after [1] was published.
- The article cited as Reference 37 appears to be unretrievable.

In addition, the *PLOS One* Editors have concerns about the article’s compliance with the PLOS Authorship policy.

In light of the cumulative issues, the *PLOS One* Editors issue this Expression of Concern. Readers are advised to interpret the article [1] with caution.
