# Supplementary material for: Targeting tumor-associated genes, immune response, and circulating tumor cells in intrahepatic cholangiocarcinoma: Therapeutic potential of Atractylodes lancea (Thunb.) DC
Source: PLoS One. 2025 May 13;20(5):e0323732. doi: 10.1371/journal.pone.0323732 (PMC12074528; doi:10.1371/journal.pone.0323732)
Supplement: S1 File — (DOCX) [file pone.0323732.s001.docx]

**Table S1** The RT^2^ profiler PCR array ∆C_T_ values (mean ± SD) at Day 1 of the collected samples in each group (N = 16 per group)

| **Gene** | **Healthy** | **Group 1** | **Group 2** | **Group 3** |
| --- | --- | --- | --- | --- |
| **IL-6** | 16.172 ± 1.193 | 12.868 ± 0.900 | 11.553 ± 0.870 | 11.597 ± 1.041 |
| **IL-10** | 11.614 ± 1.337 | 12.252 ± 0.950 | 11.806 ± 0.920 | 11.451 ± 0.933 |
| **PRF1** | 17.984 ± 1.088 | 18.983 ± 0.900 | 18.516 ± 0.907 | 18.762 ± 0.984 |
| **CTLA4** | 18.479 ± 1.013 | 16.815 ± 0.607 | 16.319 ± 0.472 | 16.594 ± 0.858 |
| **VEGFA** | 18.193 ± 1.029 | 19.105 ± 0.863 | 18.718 ± 0.796 | 18.405 ± 1.079 |
| **Ki67** | 19.453 ± 0.844 | 19.560 ± 0.869 | 19.047 ± 0.468 | 19.388 ± 0.708 |
| **NR4A3** | 19.435 ± 0.814 | 19.364 ± 0.819 | 19.231 ± 0.433 | 19.400 ± 0.774 |
| **NOS2** | 18.754 ± 0.924 | 16.965 ± 0.901 | 16.344 ± 0.775 | 16.598 ± 0.874 |
| **Fas** | 18.543 ± 1.042 | 12.256 ± 0.980 | 11.351 ± 0.764 | 12.130 ± 1.157 |
| **EpCAM** | 19.435 ± 0.814 | 12.339 ± 1.182 | 12.164 ± 0.860 | 11.597 ± 1.041 |

**Table S2** The RT^2^ profiler PCR array ∆C_T_ values (mean±SD) at Day 90 of the collected samples from triplicate repeat

| **Sample**  **Gene** | **Healthy** | **Group 1** | | | **Group 2** | | | | | | | **Group 3** |
| --- | --- | --- | --- | --- | --- | --- | --- | --- | --- | --- | --- | --- |
|  |  | **1-1** | **1-2** | **1-3** | **2-1** | **2-2** | **2-3** | **2-4** | **2-5** | **2-6** | **2-7** | **3-1** |
| **IL-6** | 19.219 ± 0.110 | 15.143 ± 0.572 | 11.173 ± 0.590 | 13.180 ± 0.956 | 13.130 ± 0.201 | 13.513 ± 0.841 | 16.600 ± 1.258 | 16.080 ± 0.128 | 15.213 ± 0.427 | 16.537 ± 1.400 | 12.823 ± 0.482 | 3.150 ± 0.597 |
| **IL-10** | 14.920 ± 0.593 | 13.747 ± 0.620 | 14.780 ± 0.005 | 14.117 ± 0.005 | 13.150 ± 0.310 | 13.315 ± 2.105 | 16.750 ± 0.483 | 16.700 ± 0.150 | 15.280 ± 0.933 | 16.910 ± 0.042 | 14.267 ± 3.843 | 21.800 ± 0.555 |
| **PRF1** | 20.030 ± 0.927 | 20.437 ± 0.021 | 21.850 ± 1.280 | 19.697 ± 0.123 | 21.283 ± 0.147 | 17.580 ± 0.605 | 20.257 ± 0.511 | 19.843 ± 0.505 | 18.793 ± 1.429 | 20.207 ± 1.502 | 20.337 ± 1.426 | 18.930 ± 0.560 |
| **CTLA4** | 21.393 ± 1.927 | 21.680 ± 0.370 | 20.203 ± 0.599 | 20.277 ± 0.385 | 20.120 ± 0.213 | 13.800 ± 0.727 | 14.847 ± 0.257 | 14.350 ± 0.475 | 13.663 ± 0.898 | 13.660 ± 0.949 | 16.600 ± 3.446 | 13.433 ± 0.635 |
| **VEGFA** | 22.110 ± 0.304 | 22.283 ± 0.231 | 21.370 ± 1.047 | 21.023 ± 1.110 | 17.860 ± 1.562 | 22.240 ± 0.122 | 22.683 ± 0.468 | 22.207 ± 0.560 | 22.363 ± 0.304 | 24.180 ± 1.958 | 19.607 ± 2.522 | 13.950 ± 0.120 |
| **Ki67** | 22.110 ± 1.419 | 18.807 ± 0.709 | 19.520 ± 1.742 | 17.710 ± 0.500 | 21.390 ± 0.468 | 21.967 ± 0.528 | 22.623 ± 0.122 | 20.283 ± 1.145 | 22.317 ± 0.825 | 23.550 ± 0.126 | 22.067 ± 3.295 | 12.940 ± 0.495 |
| **NR4A3** | 21.670 ± 1.192 | 18.773 ± 0.435 | 18.880 ± 0.551 | 19.120 ± 1.095 | 21.283 ± 0.698 | 21.197 ± 0.326 | 21.757 ± 0.179 | 21.620 ± 0.500 | 22.580 ± 1.958 | 21.720 ± 0.894 | 21.700 ± 2.824 | 14.950 ± 0.150 |
| **NOS2** | 20.990 ± 1.589 | 17.877 ± 1.533 | 20.373 ± 0.645 | 18.907 ± 0.415 | 19.350 ± 1.280 | 21.977 ± 0.825 | 22.210 ± 0.126 | 22.480 ± 0.122 | 22.423 ± 0.468 | 23.907 ± 0.528 | 21.850 ± 3.672 | 13.360 ± 0.210 |
| **Fas** | 21.130 ± 1.067 | 19.567 ± 0.519 | 19.520 ± 1.429 | 17.703 ± 0.060 | 21.547 ± 0.066 | 22.083 ± 0.467 | 22.567 ± 0.529 | 22.263 ± 0.220 | 20.383 ± 1.280 | 23.917 ± 0.825 | 21.437 ± 4.457 | 11.940 ± 0.375 |
| **EpCAM** | 22.270 ± 1.301 | 18.920 ± 1.234 | 19.787 ± 1.501 | 18.490 ± 0.065 | 21.033 ± 0.639 | 21.353 ± 0.177 | 21.930 ± 0.014 | 21.873 ± 0.675 | 21.543 ± 0.401 | 22.483 ± 1.909 | 21.153 ± 3.426 | 13.390 ± 0.165 |
