# Supplementary material for: Targeting tumor-associated genes, immune response, and circulating tumor cells in intrahepatic cholangiocarcinoma: Therapeutic potential of Atractylodes lancea (Thunb.) DC
Source: PLoS One. 2025 May 13;20(5):e0323732. doi: 10.1371/journal.pone.0323732 (PMC12074528; doi:10.1371/journal.pone.0323732)
Supplement: Attachment — Submitted filename: Response To Reviewers.docx [file pone.0323732.s004.docx]

Chulabhorn International College of Medicine

Thammasat University, Rangsit Campus

Klong Luang, Pathumthani 12120

Thailand

April 2, 2024

Dear Editor,

I am submitting the revised version of the manuscript entitled “*Targeting Tumor-Associated Genes, Immune Response, and Circulating Tumor Cells in Intrahepatic Cholangiocarcinoma: Therapeutic Potential of Atractylodes lancea (Thunb.) DC.*” for consideration of publication in *“PLOS One”*. Summary of responses to reviewers’ comments is attached herewith.

**For the** “Funding Statement” section, we do need to provide this detail for all the Funding sources. Otherwise, the published work will not be counted as the output from the funding. The current Funding Statement is required “This research project was supported by the Thailand Science Research and Innovation Fundamental Fund Fiscal Year 2023 and Thammasat University (Chulabhorn International College of Medicine, Center of Excellence in Pharmacology and Molecular Biology of Malaria and Cholangiocarcinoma). Kesara Na-Bangchang is funded by the National Research Council of Thailand (NRCT), Contract number N42A671041.”

**With regard to the “Data Availability Statement”, I confirm that the submission contains all raw data required to replicate the results of the study.** All relevant data are within the manuscript and its Supporting Information files.

Thank you very much for your consideration.

Yours Sincerely,

Prof. Kesara Na-Bangchang

Graduate Studies, Chulabhorn International College of Medicine, Thammasat University (Rangsit Center).

99 Moo 18, Paholyothin Road, Klongnung, Klongluang, Pathumthani 12120, Thailand,

Tel +66 2 564 4400 ext 1800; Fax +66 2 564 4398

E-mail: nkesara@tu.ac.th

**SUMMARY FO RESPONSE TO COMMENTS**

**PONE-D-24-55645R1**

**Targeting Tumor-Associated Genes, Immune Response, and Circulating Tumor Cells in Intrahepatic Cholangiocarcinoma: Therapeutic Potential of Atractylodes lancea (Thunb.) DC.**

**PLOS ONE**

**Editor:**

Please include the following items when submitting your revised manuscript:

- A rebuttal letter that responds to each point raised by the academic editor and reviewer(s). You should upload this letter as a separate file labeled 'Response to Reviewers'.
- A marked-up copy of your manuscript that highlights changes made to the original version. You should upload this as a separate file labeled 'Revised Manuscript with Track Changes'.
- An unmarked version of your revised paper without tracked changes. You should upload this as a separate file labeled 'Manuscript'.

2.	If you would like to make changes to your financial disclosure, please include your updated statement in your cover letter. Guidelines for resubmitting your figure files are available below the reviewer comments at the end of this letter.

3.	If applicable, we recommend that you deposit your laboratory protocols in protocols.io to enhance the reproducibility of your results. Protocols.io assigns your protocol its own identifier (DOI) so that it can be cited independently in the future.

**Comments to the Author**

1.	If the authors have adequately addressed your comments raised in a previous round of review and you feel that this manuscript is now acceptable for publication, you may indicate that here to bypass the “Comments to the Author” section, enter your conflict of interest statement in the “Confidential to Editor” section, and submit your "Accept" recommendation.

Reviewer #1: All comments have been addressed

Reviewer #2: All comments have been addressed

2. Is the manuscript technically sound, and do the data support the conclusions?

The manuscript must describe a technically sound piece of scientific research with data that supports the conclusions. Experiments must have been conducted rigorously, with appropriate controls, replication, and sample sizes. The conclusions must be drawn appropriately based on the data presented.

Reviewer #1: Yes

Reviewer #2: Yes

3. Has the statistical analysis been performed appropriately and rigorously?

Reviewer #1: Yes

Reviewer #2: No

4. Have the authors made all data underlying the findings in their manuscript fully available?

The [PLOS Data policy](http://track.editorialmanager.com/CL0/http:%2F%2Fwww.plosone.org%2Fstatic%2Fpolicies.action%23sharing/1/010f0195ea023634-d20f0979-db10-4d22-93bc-d01c6bbd6666-000000/CGYpS-U1K5InJt1nbw_rLgkHJ7M-g02pkk1dwJ5Tt_g=204) requires authors to make all data underlying the findings described in their manuscript fully available without restriction, with rare exception (please refer to the Data Availability Statement in the manuscript PDF file). The data should be provided as part of the manuscript or its supporting information, or deposited to a public repository. For example, in addition to summary statistics, the data points behind means, medians and variance measures should be available. If there are restrictions on publicly sharing data—e.g. participant privacy or use of data from a third party—those must be specified.

Reviewer #1: Yes

Reviewer #2: Yes

5. Is the manuscript presented in an intelligible fashion and written in standard English?

PLOS ONE does not copyedit accepted manuscripts, so the language in submitted articles must be clear, correct, and unambiguous. Any typographical or grammatical errors should be corrected at revision, so please note any specific errors here.

Reviewer #1: Yes

Reviewer #2: Yes

6. Review Comments to the Author

Please use the space provided to explain your answers to the questions above. You may also include additional comments for the author, including concerns about dual publication, research ethics, or publication ethics. (Please upload your review as an attachment if it exceeds 20,000 characters)

Reviewer #1: The reviewers have adequately addressed my comments on the manuscript 'Targeting Tumor-Associated Genes, Immune Response, and Circulating Tumor Cells in Intrahepatic Cholangiocarcinoma: Therapeutic Potential of Atractylodes lancea (Thunb.) DC.' and I propose the acceptance of the article.

Reviewer #2: The authors have significiantly reworked the manuscript. The conclusion has been adequately toned down to fit the results. The limitations and further research requirements have been outlined well. It is unfortunate, that a study with such an interesting premise is so strongly impaired by a high loss to follow up from an already small sample size - yet, this is hard to avoid in terminal cancer studies.

I believe that publication is warranted, particularly as this is clinical data, after addressing two major concerns:

1) Line 171 - 172: No correction for multiple comparison is detailed here. The local alpha niveau is thus likely inflated to values far higher than 0.05 due to alpha accumulation. Please detail 1) whether your workflow already contained correction for multiple testing, as is the standard in many statistics programs including PRISM, 2) whether you performed multiple testing corrections yourself, such as the Bonferroni-Holm method, or or possibly limited the false discovery rate, or 3) that the study did not correct for alpha accumulation and why.

***Response:*** Thank you for this valuable comment. We have revised the statement in the manuscript: “Multiple testing corrections were applied using PRISM, which computes multiplicity-adjusted p-values based on the Bonferroni correction method.”

2) Figure 4: I struggle to understand, how samples were selected for this figure, as opposed to all patients in figure 2. As I understand this Figure, only samples were considered, for which data was available on day 1 and 90. However, this does not explain, how the 3 healthy subjects were selected for figure 4, as the methods section specifies they were only sampled once. Figure 4 is central to this study and would thus especially benefit from a clearer description both in the legends, as well as the results section.

***Response:*** Thank you for your insightful comment. You are correct that the healthy subjects were sampled only once, as we assumed they would not exhibit significant changes in the evaluated markers over time. For Figure 4, three samples from the healthy group were randomly selected to serve as controls for the PCR array analysis. These results were then used as a reference for comparison in Figure 4. We will clarify this selection process in both the figure legend and the results section to enhance transparency.

*Minor comments:*

1) Line 78-79: This sentence sounds like a citation is missing.

***Response:*** The references have been added.

2) Line 115-116: "is a capsule formation of"

***Response:*** This has been corrected.

3) Line 131: "enrollment".

***Response:*** This has been corrected.

4) Line 132 - 133: The conversion of RPM to G differs substantially by centrifuge design, i.e., the radius of the rotor. It would be highly beneficial for anyone attempting to reproduce this study, if the acceleration (in xG) was also provided. Alternatively, the authors could provide the exact model of the centrifuge and rotor, so that others can calculate the G-force.

***Response:*** The sentence has been revised as “Blood samples were centrifuged at 9,000-12,000 *xg* for 5 min to separate blood components.”

5) Line 169: "are summarized as number (%)" I'm not perfectly sure what the authors mean by this, but I assume the authors refer to the number and percent of the population fulfilling or not fulfilling a qualitative criteria.- "Patients sampled" or "Patients available" may be more appropriate than formulating "Patients used".

***Response:*** The sentence has been revised as “Qualitative data are summarized as the number (%) of patients available.”

6) Line 177: The abstract details collection of 59 samples, this sentence mentions 64 samples.

***Response:*** The number has been corrected as 59 samples.

7) Table 1: It's great that the authors added this table. For quick visual assessment, the authors may consider also adding "(n,%)" over the first row of data of that table (e.g., second cell, left column). I also believe that they're likely referring to the Karnofsky (not "Kamafsky") Performence Index.

***Response:*** The column has been added as suggested and the word ‘Karnofsky’ has been corrected.

8) Line 297-298: The authors don't seem to use this system (Methods), so this sentence my be irrelevant.

***Response:*** The sentence has been removed.

9) Line 356: Was this Group treated with Placebo? The methods section introduces it as "best supportive care" group.

***Response:*** This has been corrected as ‘control group’ (supportive treatment alone).

10) 371 - 372: This statement is also made in the sentence prior. As the authors do not analyse immune function as part of this study, this conclusion is likely overdrawn, except if linked to previous work showing functional differences of the immune response.

***Response:*** This statement has been removed.

11) Line 375- 376: I appreciate the authors' well-written and comprehensive limitation statement. The sentence ending in "...cannot be concluded." is understandable, but doesn't read well. Consider something like "Therefore, it is unclear whether any observed changes [...] translate to differences in protein expression or cell function."

***Response:*** The sentence has been revised as “Therefore, it is unclear whether any observed changes in gene expression due to circulating immune cells cannot be concluded.”

Additional suggestions:

12) Line 282 - 306: The general merit of liquid biopsies and CTC could be explained with much more brevity, to make room for a more thorough discussion of the actual results, as they relate to other studies in the field.

***Response:*** This has been revised as “Liquid biopsies, including the detection of circulating tumor cells (CTCs) and circulating invasive cells (CICs), have emerged as promising non-invasive tools for cancer diagnosis, prognosis, and treatment monitoring. Unlike traditional tissue biopsies, liquid biopsies enable real-time tracking of tumor evolution, facilitating early detection of recurrence, treatment resistance, and minimal residual disease. Their minimally invasive nature reduces the risk and discomfort associated with conventional biopsy procedures, making them a valuable approach for longitudinal monitoring in clinical practice [37].”

7. PLOS authors have the option to publish the peer review history of their article ([what does this mean?](https://track.editorialmanager.com/CL0/https:%2F%2Fjournals.plos.org%2Fplosone%2Fs%2Feditorial-and-peer-review-process%23loc-peer-review-history/1/010f0195ea023634-d20f0979-db10-4d22-93bc-d01c6bbd6666-000000/HJ6uw1YdxY-YUujLvg1H482UQK2g97tpZe6u_Azw7yc=204)). If published, this will include your full peer review and any attached files.


If you choose “no”, your identity will remain anonymous but your review may still be made public.


**Do you want your identity to be public for this peer review?** For information about this choice, including consent withdrawal, please see our [Privacy Policy](https://track.editorialmanager.com/CL0/https:%2F%2Fwww.plos.org%2Fprivacy-policy/1/010f0195ea023634-d20f0979-db10-4d22-93bc-d01c6bbd6666-000000/bSTF2gNcHODxUpA7-xTmrPJD2357ooHlzeQAPqVEivU=204).

Reviewer #1: **Yes:** Mohamed Abdelkarim

Reviewer #2: **Yes:** Willem Buys
